# Insights into Supplements with Tribulus Terrestris used by Athletes

**DOI:** 10.2478/hukin-2014-0037

**Published:** 2014-07-08

**Authors:** Andrzej Pokrywka, Zbigniew Obmiński, Jadwiga Malczewska-Lenczowska, Zbigniew Fijałek, Ewa Turek-Lepa, Ryszard Grucza

**Affiliations:** 1 Department of Anti-Doping Research, Institute of Sport, Warsaw, Poland.; 2 Department of Endocrinology, Institute of Sport, Warsaw, Poland.; 3 Department of Nutrition Physiology, Institute of Sport, Warsaw, Poland.; 4 Pharmaceutical Chemistry Department, National Medicines Institute, Warsaw, Poland.; 5 Department of Exercise Physiology, Collegium Medicum in Bydgoszcz, Nicolaus Copernicus University, Poland.

**Keywords:** Sports, dietary supplements, doping, placebo, testosterone, herbal supplements

## Abstract

Herbal and nutritional supplements are more and more popular in the western population. One of them is an extract of an exotic plant, named Tribulus terrestris (TT). TT is a component of several supplements that are available over-the-counter and widely recommended, generally as enhancers of human vitality. TT is touted as a testosterone booster and remedy for impaired erectile function; therefore, it is targeted at physically active men, including male athletes. Based on the scientific literature describing the results of clinical trials, this review attempted to verify information on marketing TT with particular reference to the needs of athletes. It was found that there are few reliable data on the usefulness of TT in competitive sport. In humans, a TT extract used alone without additional components does not improve androgenic status or physical performance among athletes. The results of a few studies have showed that the combination of TT with other pharmacological components increases testosterone levels, but it was not discovered which components of the mixture contributed to that effect. TT contains several organic compounds including alkaloids and steroidal glycosides, of which pharmacological action in humans is not completely explained. One anti-doping study reported an incident with a TT supplement contaminated by a banned steroid. Toxicological studies regarding TT have been carried out on animals only, however, one accidental poisoning of a man was described. The Australian Institute of Sport does not recommend athletes’ usage of TT. So far, the published data concerning TT do not provide strong evidence for either usefulness or safe usage in sport.

## Introduction

Nowadays, modern pharmacology is based mostly on synthetic chemical compounds. However, traditional herbal medicine which utilizes centuries-old experience, still plays an important and complementary role in enhancement of human health. The pharmaceutical market advertises and provides several products, so-called nutritional and dietary supplements, which are not considered as drugs; therefore, their quality and biomedical efficacy are not required to be strictly controlled by clinical trials. Nevertheless, herbal extracts containing biologically active chemical compounds serve sometimes as components of those supplements. The use of herbal extracts or nutritional supplements containing those components is becoming more and more attractive and popular among the Western population due to extensive marketing activities. However, there is a need to confirm the recommended usefulness and possible unknown side effects when using herbs, especially those that contain biologically active organic chemicals. It may be achieved due to advanced and trustworthy scientific methodologies of clinical trials, which allow one to verify the former attitudes and beliefs referring to the effectiveness of the action of herbal preparations. One of the many exotic herbal plants recommended for the use is *Tribulus terrestris* (TT), which was used by traditional, ancient medicine in Greece, China and India (Ayurvedic medicine). It was recommended as a remedy for infertility, impotence, erectile dysfunction, and low libido. Since the early 1980s its extract has also been an attractive product of unconventional medicine in Western countries as a testosterone booster, an enhancer of libido and a adaptogenic aid for healthy and physically active men. Concurrently, physical activity in leisure time is widely recommended by WHO for prevention of lifestyle diseases, good performance and wellbeing; therefore, proper diet and the use of nutritional supplements, including herbs, can also be effectively advertised. TT as a testosterone enhancer would be attractive also for competitive athletes, as an alternative of the use of forbidden enhancing drugs such as androgenic-anabolic steroids. No wonder that recently TT promotion has been targeted in a high extent to athletes. An additional incentive and facilitation for the use is that the extract of TT is provided by the pharmaceutical market as an over-the-counter supplement.

## Aim of the review

Taking into consideration an increasing interest in the extract of TT as a supplement improving human vitality, the purpose of this review was to present current knowledge of the effects of the use of TT on androgenic status and physical performance in healthy, physically active men, with special focus on the benefits and risks for human health.

## Characteristics of TT

The plant grows mainly in Africa, Asia, Australia and Europe. It has seeds with three spines and yellow flowers, and grows up to a metre high. For a long time it was believed that besides the special biological properties which make this plant attractive as an enhancer of human vitality, all the components of the plant, roots, seeds, fruit and leaves, are useful in cases of kidney stones, high cholesterol, hypertension and as a diuretic. Currently, TT is mainly advertised as a testosterone booster, that may be a way to improve androgenic status in case of male hypogonadism and may enhance performance in athletes. The majority of these assumptions are provided by the Internet and other media which aim to encourage the use of TT. Usually, TT extracts are offered by the pharmaceutical market as a separate product, or as a component of various nutritional supplements, which are offered for healthy, physically active adults, athletes and generally for those who want to maintain their health and well-being. However, in spite of alleged benefits, potential users should get to know, and take into consideration, the results of clinical trials, which are often not clear, and some of them are not fully credible because of methodological shortcomings. In fact, the effectiveness of several herbal supplements examined in a controlled experimental setting is often significantly lower compared to common opinions of the users, especially those of higher susceptibility to suggestions. All those doubts as to the effectiveness of TT have to be verified. The present review focused on currently available scientific and trustworthy information about potential advantages and side effects resulting from the persistent taking of TT, and a discussion of the rationale of TT usage for revitalization by improving androgenic status in athletes. Scientific literature referring to this topic is limited, and the results of existing studies do not present clear conclusions.

Analysis of the chemical composition of *Tribulus terrestris* revealed contents of many chemical compounds, among which the best known are steroidal glycosides (saponins) and alkaloids. Presumably, so far, not all chemical compounds have been identified in the plant, since researchers are still discovering new substances of a high molecular weight which belong to the family of steroidal saponins, glycosides, alkaloids and flavonoids. The majority of those compounds have not been identified earlier by chemists; therefore, their new specific names come from the name of the plant (terrestribisamide, tribulusterine, terrestrosin D) (Chhatre et al., 2014). The quantitative content of those compounds is not stable, but dependent on the climate and geographical region (Dinchev et al., 2008).

## Use of TT in sport. A risk of doping

First scientific reports about beneficial effects resulting from the use of TT were provided by researchers of the Bulgarian Pharmaceutical Group. They stated that TT extracts increased libido, blood testosterone and spermatogenesis, and improved the male sexual function. To date, there is a lack of reliable data concerning the usefulness and safe usage of TT in sport. However, TT is widely touted among athletes and used by them. Ergogenic (anabolic) properties are attributed to this plant, since it supposedly elevates the blood testosterone level and stimulates skeletal muscles hypertrophy. TT was especially promoted in Bulgaria, where it was used from the 1970s as the preparation named Tribestan (Bucci, 2000; Koumanov et al., 1982). It was suggested that improvements in physical performance among Bulgarian athletes, especially weightlifters, were achieved due to the use of this product.

Indeed, that was a time when Bulgarian weightlifters were the world champions, until more accurate anti-doping controls revealed the use of anabolic-androgenic steroids in these athletes. Hence, until the absence of steroidal doping will be confirmed by urine analysis, good sport results in weightlifting should be taken with caution. Despite discovered doping cases among Bulgarian weightlifters during the Olympic Games (Predergast et al., 2003) they did not stop using doping. Because of the disqualification of 11 doping users, the Bulgarian weightlifting team was excluded from the Olympic Games in Beijing (2008).

Summing up, there is no strong evidence for the contribution of TT to success in weightlifting. In response to aggressive marketing of nutritional supplements which are to improve health and physical performance, it is worth noting that nutritional supplements which are recommended for competitive athletes to enhance their performance may be contaminated by androgenic-anabolic steroids (AAS) and so-called pro-hormones that are weak androgens, which are precursors of stronger endogenous androgens such as testosterone and dihydrotestosterone (DHT). Such contamination is not usually described in the list of ingredients; however, on the market there also exist products with a full list of components, including compounds prohibited in sport. Moreover, some researchers undertake studies to examine the biological action of those compositions, i.e. TT plus AAS. In the search for appropriate androgens taken by athletes, Brown et al. (2000b) focused their attention on exogenous androstenedione and androstenediol, steroids which in the human body are converted into more biologically active compounds. Assuming that TT would enhance the rate of that conversion, and in consequence increase the effectiveness of relatively small amounts of those pro-hormones, the same authors carried out more comprehensive studies on the biological effect of TT taken concurrently with androstenediol (Brown et al., 2001) or androstenedione (Brown et al., 2000a). The TT-androgen compositions were tested among healthy males aged 30–59 years receiving an extract of TT with 100 mg of pro-hormone/day. The results did not show changes in blood total testosterone in both cases, but a significant increase (by 37%) of free, biologically active fraction of testosterone induced by androstenediol–*Tribulus terrestris* supplementation (Brown et al., 2001), and practically unchanged free testosterone following ingestion of androstenedione–*Tribulus terrestris* composition (Brown et al., 2000a). However, when a daily dosage of oral ingestion of androstenedione was much higher (300 mg) without TT, young men improved their muscle strength after an 8-week strength training period, and their free testosterone increased by 45% (King et al., 1999). The mentioned type of experiments indicate that some studies are focused on a search of herbal-androgenic compositions which would have anabolic action despite a relatively small (un-detected) amount of banned androgens. However, such procedures may suggest that extracts of TT are not as effective as the users, mostly athletes, expect. The same is true regarding other nutritional supplements recommended for athletes. For that reason, it is no wonder that some supplements are contaminated intentionally by banned substances which may enhance athletic performance (Aqai et al., 2013; Cavalcanti Gde et al., 2013; Judkins and Prock, 2012). Obviously, the contamination of nutritional supplements may lead to inadvertent doping in competitive sport. One of the most spectacular incidents of non-intentional doping was recorded just prior to the Olympic Games, when the Norwegian weightlifter Stian Grimseth was disqualified for taking nutritional supplements containing ribose, but contaminated by non-listed 19-norandrostenedione, as was described in 2011 by the quarterly magazine World Weightlifting. Since TT is often used by athletes it is worth knowing whether taking TT extract may change the urinary endogenous androgenic profile to give a positive result of an anti-doping test. There are few studies carried out among athletes to elucidate the degree of risk of taking TT. The results showed that taking TT without any contaminations did not cause positive anti-doping tests (Saudan et al., 2008; Van Eenoo et al., 2000). Despite this, some scientists state that adequate controls of TT purity are still lacking. The Australian Institute of Sport (AIS) has developed a Sports Supplements Program which was designed for the specific needs of AIS and other Australian athletes. Among other aspects, this program aims to allow AIS athletes to focus on the use of supplements and specific sports diet as part of their nutrition plans and to minimize the risk of the supplement use leading to an inadvertent doping offence. In this program supplements are classified into four groups according to their effectiveness and safety. TT as well as other testosterone boosters are included in the group D named “Banned or at high risk of contamination”. Compounds from this group should not be used by athletes (AIS, 2014). That risk of the contaminated TT extracts appeared to be warranted because of detection of AAS (4-androstene-3,17-dion, 4-androstene-3ß,17ß-diol, 5-androstene-3ß,17ß-diol, 19-nor-4-androstene-3,17-dion and 19-nor-4-androstene-3ß,17ß-diol) in TT products. These steroids were not listed on the label (Geyer et al., 2000).

## Does TT really work in humans?

Studies on effects of TT on blood androgens and a sexual disposition were conducted mainly among animals, and the results were contradictory. As to effects on the libido level, the experiments carried out in castrated rats showed that an extract of TT administered orally (5 mg/kg) possesses aphrodisiac activity (Gauthaman et al., 2002), and the effects were dose-dependent (Singh et al., 2012). Studies carried out in primates (baboons and rhesus monkeys), rabbits and castrated rats showed that acute intravenous treatment with a TT extract (7.5 mg/kg) resulted in a significant rise of blood androgens – testosterone, DHT and DHEAS – by 52, 31 and 29% respectively in primates, while in rabbits DHT increased by 30%, and in castrated rats total testosterone in blood was elevated by 25% (Gauthaman and Ganesan, 2008). Similar results reported by El-Tantawy et al. (2007) showed significantly higher levels of serum free fraction of testosterone in rats treated throughout a 40-day period with extracts of TT. In contrast to those results, the study carried out by Martino-Andrade et al. (2010) showed no changes in blood status of testosterone in castrated rats following a 28-day period of oral treatment with a high daily dosage of TT. On the other hand, there was evidence that rats concurrently receiving TT and morphine presented significantly lower reduction of blood sex hormones and pituitary gonadotropin (luteinizing hormone), in comparison to those exposed only to the narcotic (Ghosian Moghaddam et al., 2013).

A few studies with a TT extract carried out among humans also provided divergent results. Since for the pharmaceutical market, which provides TT extracts, the main targets are athletes and healthy, physically active adults, the whole investigative efforts are aimed at confirming such biomedical properties of TT that are expected by the potential users. As mentioned, athletes seek performance enhancers, i.e. supplements of ergogenic properties, to improve training tolerance, and increase muscle mass and physical strength or endurance. Since development of muscle mass and strength depends, in part, on androgenic status, competitive athletes seek substances that are able to increase their endogenous circulating testosterone, while guaranteeing a negative anti-doping test. So far, trustworthy studies on expected TT properties are still insufficient. Neychev and Mitev (2005) found that neither a daily lower (10 mg/kg) nor a higher dose of TT (20 mg/kg) taken orally had an effect on blood testosterone, androstenedione or luteinizing hormone following a 4 week period of supplementation. Van Eeenoo et al. (2000) reported no changes in blood testosterone and LH following a 5 day period of supplementation of TT (750 mg/day), and an unchanged urinary testosterone level to the epitestosterone ratio. Likewise, other studies conducted among athletes did not confirm the beneficial effect of supplementation of TT on physical performance. Resistance-trained men who received a daily dose of TT amounting to 3.2 mg/kg during an 8-week training period did not improve their results of bench and leg press, mood states, body mass and composition did not change (Antonio et al., 2000). A higher TT daily dosage (450 mg/day) taken by rugby players over a 5 week training period also did not cause changes in strength, body composition and the urinary testosterone/epitestosterone ratio (Rogerson et al., 2007). In contrast to these data, there are two studies confirming beneficial effects after treatment with pharmaceutical products containing TT and other components. After 20 day supplementation with the dietary supplement “Tribulus” anaerobic and alactic muscular power and blood testosterone significantly increased among youth men (Milasius et al., 2009). The other placebo-controlled double-blind experiment conducted among older men with formerly impaired erectile function and lowered blood total (8.0 nmol/L) and free (0.19 nmol/L) testosterone levels showed very high effectiveness of a preparation containing TT. This product, named “Tradamixina”, being a composition of TT, Alga Eckonia, D-glucosamine and N-acetylglucosamine, administered every day, throughout a 2 month period, improved libido and elevated the testosterone fractions to the average levels of 23.3 and 0.42 nmol/l, respectively (Iacono et al., 2012). It should be stressed, however, that in both experiments, there was no certainty which component(s) of those products caused the biological advantages, and whether TT contributed to those effects.

## Side effects

Studies on TT toxicity have been conducted only among animals. Arcasoy et al. (1998) established for mice that the dose corresponding LD 50 amounts to 813 mg/kg. Symptoms of severe damage of cardiac muscle, liver and kidney were noted in native goats and sheep when their daily meals contained 80% fresh plants (Aslani et al., 2003; Aslani et al., 2004). The only case of acute poisoning by TT was reported in a young man, who consumed during two days a high dose of TT to prevent kidney stone formation. He was hospitalized, and after 7 days biochemical symptoms of hepatitis and kidney necrosis were decreased (Talazas et al., 2010). As was shown, potential benefits and risks for human health as a result of supplementation of TT still remain unclear.

## Herbs and nutritional supplements in sports in the future

Effects of the use of nutritional supplements in sports are widely studied. The results of numerous investigations referring to the biological action, duration of supplementation and recommended doses are currently published in the scientific literature (Czeczelewski et al., 2013; Desbrow et al., 2012; Helms et al., 2014; Patlar et al., 2012; Ranchordas et al., 2013; Roshan et al., 2013; Santos et al., 2012; Seferoğlu et al., 2012), and this information is sometimes listed on the label; therefore, sport physicians, coaches and athletes usually know how to use those products. Yet, knowledge of the physiological action of exotic herbs among sport physicians seems to be inadequate in the light of the large number of easily available over-the-counter new herbal products accompanied by a limited number of clinical trials and up-to-date information. For that reason, there are concerns that some herbs do not work or, what is worse, may cause unpredicted adverse effects as an alone product, or disadvantageous herb-drug interactions (Canter and Ernst, 2004; Izzo, 2012). This refers especially to TT extracts containing biologically active chemical compounds such as alkaloids and glycosides.

## Conclusions and recommendations

Considering a relatively low number of conducted studies on the influence of TT on athletes, especially with regard to the effect on performance and androgenic status, one should stress the lack of evidence to show an enhancing effect of the use of TT on expected biomedical properties in humans. Contradictory results of aforementioned studies suggest that marketing opinions about TT as a testosterone enhancer are unsubstantiated. Therefore, there are athletes who use it in order to improve their performance. It is caused by intensive advertising encouraging TT usage, which may only result in a temporary placebo effect. For that reason, further clinical trials should be carried out in the future.
